# Haemosporidian Parasites of Antelopes and Other Vertebrates from Gabon, Central Africa

**DOI:** 10.1371/journal.pone.0148958

**Published:** 2016-02-10

**Authors:** Larson Boundenga, Boris Makanga, Benjamin Ollomo, Aude Gilabert, Virginie Rougeron, Bertrand Mve-Ondo, Céline Arnathau, Patrick Durand, Nancy Diamella Moukodoum, Alain-Prince Okouga, Lucresse Delicat-Loembet, Lauriane Yacka-Mouele, Nil Rahola, Eric Leroy, Cheikh Tidiane BA, Francois Renaud, Franck Prugnolle, Christophe Paupy

**Affiliations:** 1 Centre International de Recherches Médicales de Franceville (CIRMF), Franceville, Gabon BP 769 Franceville, Gabon; 2 Laboratory of Evolutionary Biology, Ecology and Management of Ecosystems, Faculty of Sciences and Techniques, Cheikh Anta Diop University of Dakar, Dakar, Senegal; 3 Laboratoire MIVEGEC, UMR 224–5290 IRD-CNRS-UM, Centre IRD de Montpellier, 34295 Montpellier, France; 4 Institut de Recherche en Ecologie Tropicale, Libreville, Gabon; Institute of Tropical Medicine, JAPAN

## Abstract

Re-examination, using molecular tools, of the diversity of haemosporidian parasites (among which the agents of human malaria are the best known) has generally led to rearrangements of traditional classifications. In this study, we explored the diversity of haemosporidian parasites infecting vertebrate species (particularly mammals, birds and reptiles) living in the forests of Gabon (Central Africa), by analyzing a collection of 492 bushmeat samples. We found that samples from five mammalian species (four duiker and one pangolin species), one bird and one turtle species were infected by haemosporidian parasites. In duikers (from which most of the infected specimens were obtained), we demonstrated the existence of at least two distinct parasite lineages related to *Polychromophilus* species (i.e., bat haemosporidian parasites) and to sauropsid *Plasmodium* (from birds and lizards). Molecular screening of sylvatic mosquitoes captured during a longitudinal survey revealed the presence of these haemosporidian parasite lineages also in several *Anopheles* species, suggesting a potential role in their transmission. Our results show that, differently from what was previously thought, several independent clades of haemosporidian parasites (family Plasmodiidae) infect mammals and are transmitted by anopheline mosquitoes.

## Introduction

The order Haemosporida (also called Haemosporidia, Haemospororida or Haemospororina) includes many protozoan parasites among which the best known are the agents of human malaria, a disease affecting every year several millions of people in tropical regions and particularly in sub-Saharan Africa [1]. Within the Haemosporida order, the large family of Plasmodiidae comprises several genera and species that parasitize a wide range of vertebrates, such as fish, reptiles, birds and mammals [2]. All the parasite species that infect mammals belong to this family and are restricted to nine genera (*Biguetiella*, *Bioccala*, *Dionisia*, *Nycteria*, *Hepatocystis*, *Plasmodium Polychromophilus*, *Rayella* and the fossil genus *Vetufebrus*). Although most of these parasites were described during the second half of the twentieth century, many new taxa have been discovered in wildlife during the last decade [2, 3].

The first taxonomic descriptions of haemosporidian parasites were based on morphological characteristics [4, 5] and relied mainly on the microscopic observations of biological features [6], such as the schizont and gametocyte forms [7–9], and on their life history traits as well as the nature of the host species. This approach was very useful; however, it is not very reliable [10]. Indeed, over time, the morphology of an organism can be modified by the influence of environmental factors [10]. Moreover, the morphological features and life history traits of a parasite species can vary from one host species to another [11, 12]. In addition, some species can display the same morphological features, although they are genetically very different (cryptic species) [10]. Therefore, most taxonomic identifications and classifications are now based on the species genetic characteristics obtained using molecular tools [10]. Particularly, molecular analyses of haemosporidian species have led to major rearrangements of the traditional classifications. For instance, molecular studies on avian malaria parasites revealed much more diversity than previously assumed based on morphological analyses [13, 14]. Molecular tools also revealed the existence of an abundant diversity of *Plasmodium* species that infect African great apes [15–18]. This diversity was largely underestimated by microscopic examinations [19].

Recently, following a longitudinal survey of sylvan anopheline mosquitoes in Gabon (Central Africa), we discovered several phylogenetic lineages of haemosporidian parasites (based on the sequence characterization of a cytochrome B fragment in the mitochondrial genome) for which the closest reference sequence in Genbank was a sequence obtained from an African monkey (isolate S2138 [20].). Besides this sequence, the closest reference sequences were from parasites infecting birds (e.g. *Plasmodium* sp_GD2_GD201). Despite significant screening of malaria parasites in central African monkeys, we could not find any other sequence related to S2138 in monkeys [20, 21], suggesting that monkeys are accidental hosts. Therefore, to identify the natural vertebrate hosts of these different lineages, we decided to explore or re-explore, with molecular tools, the diversity of haemosporidian parasites in the different groups of sylvatic vertebrates (from reptiles to birds and mammals) present in Gabon (excluding non-human primates and bats, the parasite diversity of which was recently revised in Central Africa; see for instance [6, 15, 20–23]). We could identify several haemosporidian lineages that infected different groups of vertebrates, especially ungulates, and that did not correspond to any of the previously described parasite species. Most of them also infected different sylvan anopheline mosquito species. Their discovery and the phylogenic position of these new lineages bring new information on the evolution of haemosporidian parasites in general and particularly on those infecting mammals. Indeed, our results show that, contrary to what previously thought (e.g., [24–26]), extant malaria parasites colonized and radiated in mammals and anopheles several times independently.

## Results and Discussion

**Fig 1:**
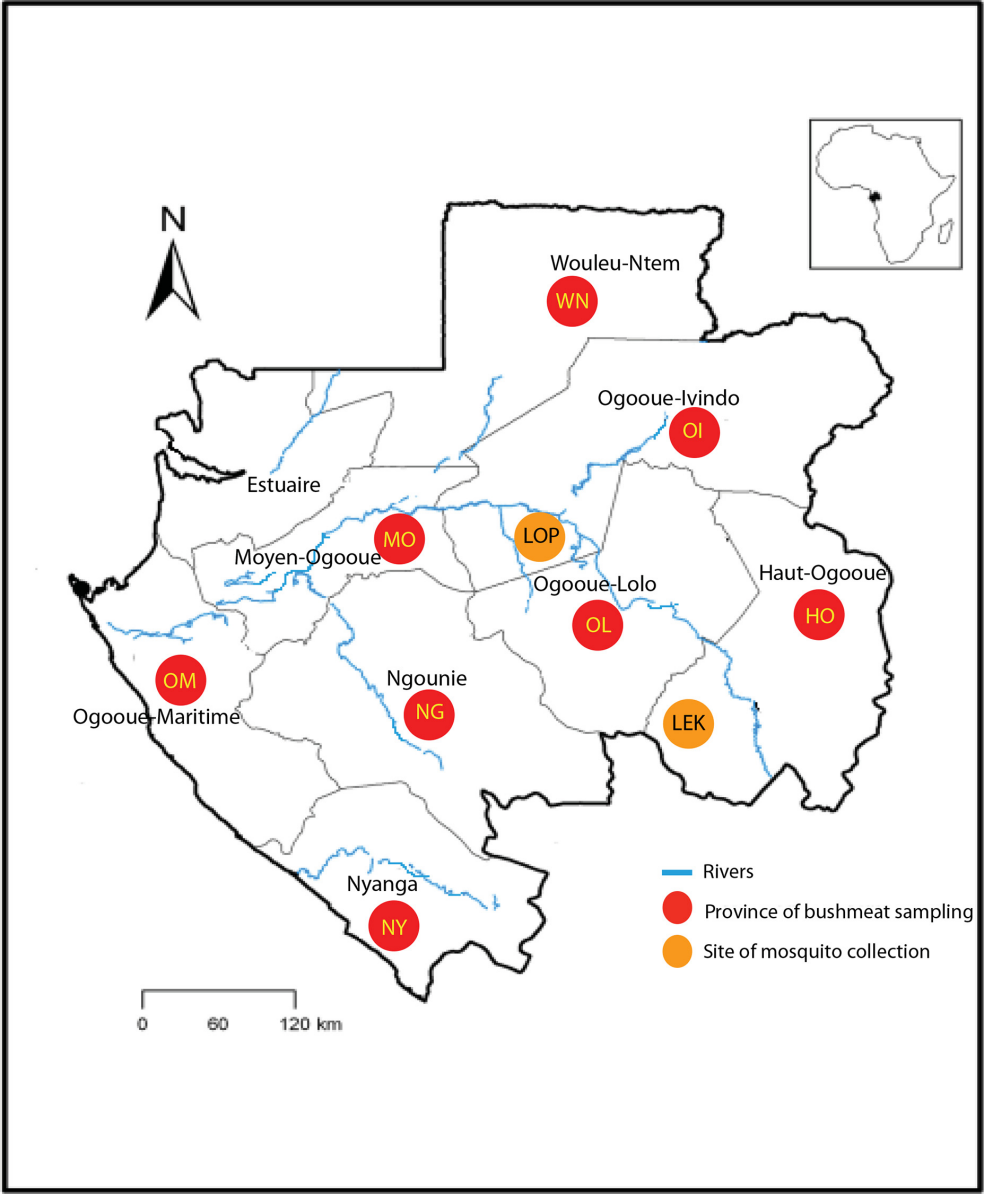
Location of the Gabon provinces where bushmeat samples were collected (OL: Ogooue-Lolo; OM: Ogooue-Maritime, OI: Ogooue-Ivindo, NG: Ngounie; NY: Nyanga; HO: Haut-Ogooue; MO: Moyen-Ogooue; WN: Woleu-Ntem) and of the two wildlife reserves where the longitudinal survey of sylvan *Anopheles* was carried out (LOP: La Lopé and LEK: La Lékédi).

**Fig 2:**
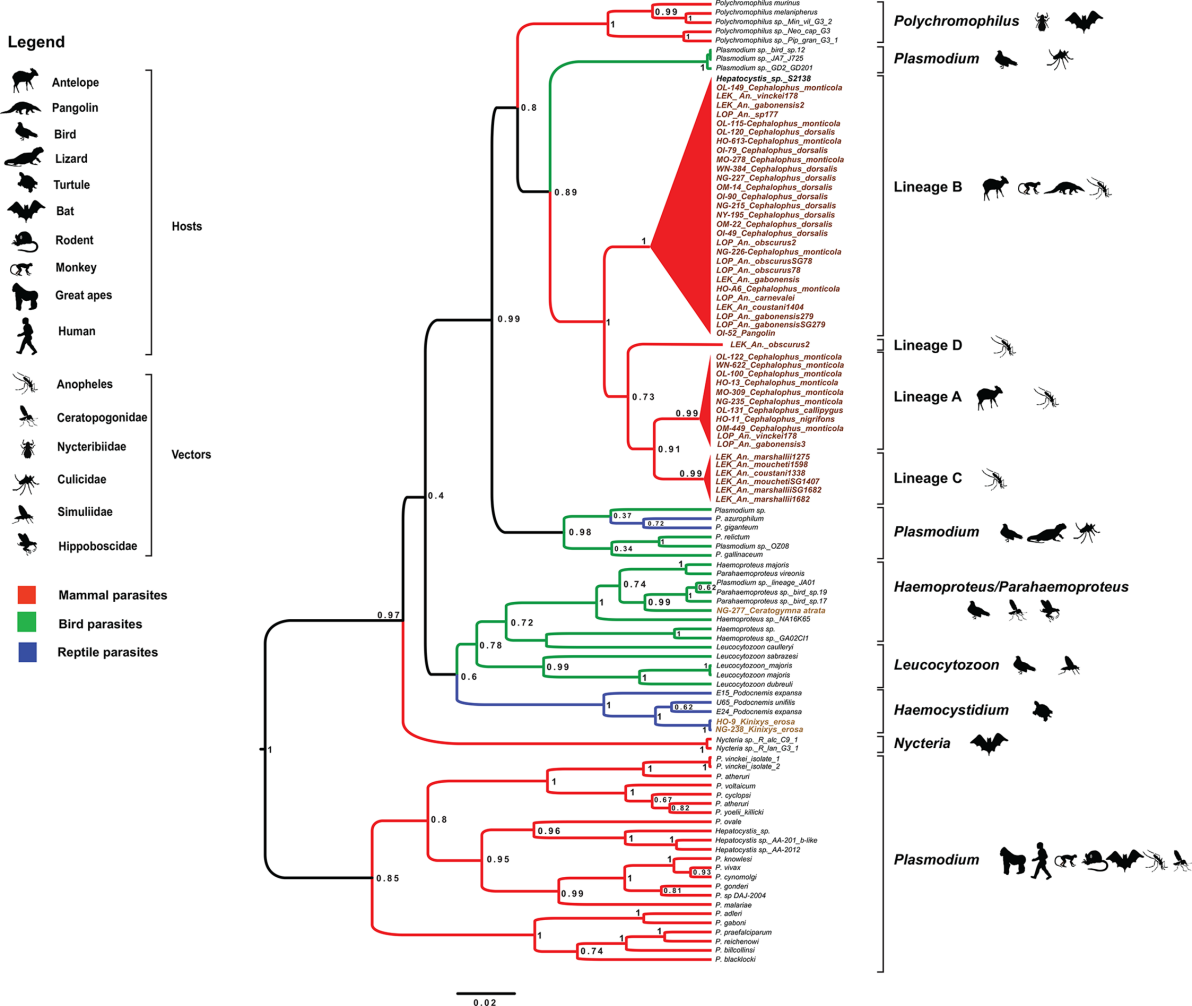
Phylogenetic relationships between the *Cyt-b* sequences of haemosporidian parasites obtained in our study and reference *Cyt-b* sequences (names in black) from existing databases. The tree was constructed using a Bayesian outgroup-free method with relaxed molecular clock assumptions. The names of our isolates include: 1) the abbreviation of the sampling site (LOP: La Lopé and LEK: Lékédi) or province (OL: Ogooue-Lolo; OM: Ogooue-Maritime, OI: Ogooue-Ivindo, NG: Ngounie; NY: Nyanga; HO: Haut-Ogooue; MO: Moyen-Ogooue; WN: Woleu-Ntem), 2) the sample number, 3) the name of the host species in which it was found (vertebrate host or anopheles) (for instance, OL115-*Cepholophus monticola*), 4) SG if the parasite was detected in the salivary glands (for anopheles only). The tree was built based on 757 bp-long *Cyt-b* sequences. Branch colors indicate different groups of vertebrates. Posterior probabilities are given at each node. More details on the different reference sequences can be found in Table A in S1 File.

In this study, we investigated the diversity of haemosporidian parasites that circulate among wild vertebrates in Gabon, Central Africa, using molecular tools to analyze the parasite content of bushmeat samples collected in different areas of Gabon (Fig 1) and stored in a biobank. This biobank included bushmeat samples from 13 species of mammals, four species of reptiles and three species of birds that represented mainly species hunted and consumed in Gabon (Table 1). Molecular analyses revealed that different species of mammals, reptiles and birds were infected by haemosporidian parasites (Table 1). Among these specimens, ungulates from the genus *Cephalophus* were infected by parasites belonging to at least two main and distinct phylogenetic Haemosporida lineages (Fig 2) (hereafter, referred as lineage A and B). We detected lineage A in three ungulate species [*Cephalophus monticola* (blue duiker), *Cephalophus calliphygus* (Peter’s Duiker) and *Cephalophus nigrifrons* (black fronted duiker)] and lineage B in two species of duikers [*C*. *monticola* and *Cephalophus dorsalis* (Bay duiker)] and one pangolin sample (*Phataginus tricuspis*). Lineage B also included one reference sequence obtained from an African monkey of the genus *Cercopithecus* (S2138) [20].

**Table 1:** Host species screened for the presence of haemosporidian parasites, number of tested vertebrate samples and number of specimens harboring a parasitic cytochrome B (*Cyt-b*) gene sequence and parasite lineage.

Vertebrate class	Common Name	Host species	Tested specimens	*Cyt-b*-positive specimens sequences obtained	Haemosporidian lineage (n. of positive samples)
**Mammals**	**Peters’s duiker**	*Cephalophus callipygus*	**19**	**1**	**Lineage A (1)**
	**Black-fronted duiker**	*Cephalophus nigrifrons*	**6**	**1**	**Lineage A (1)**
	**Blue duiker**	*Cephalophus monticola*	**170**	**13**	**Lineage A (7); Lineage B (6)**
	**Bay duiker**	*Cephalophus dorsalis*	**59**	**12**	**Lineage B (12)**
	**Pangolin**	*Phataginus tricuspis*	**38**	**1**	**LineageB (1)**
	**Sitatunga**	*Tragelaphus spekei*	**10**	**-**	
	**Red river hog**	*Potamochoerus porcus*	**10**	-	-
	**African brush-tailed porcupine**	*Atherurus africanus*	**68**	-	-
	**African civet**	*Civettictis civetta*	**8**	-	-
	**African palm civet**	*Nandinia binotata*	**25**	-	-
	**Water chevrotain**	*Hyemoschus aquaticus*	**9**	-	-
	**African bush squirrel**	*Paraxerus poensis*	**6**	-	-
	**Gambian pouched rat**	*Cricetomys gambianus*	**10**	-	-
**Birds**	**Black guineafowl**	*Agelastes niger*	**10**	-	-
	**Black-casqued hornbill**	*Ceratogymna atrata*	**9**	**1**	***Haemoproteus-like* (1)**
	**African collared dove**	*Streptopelia roseogrisea*	**2**	**-**	**-**
**Reptiles**	**Forest hinge-back tortoise**	*Kinixys erosa*	**14**	**2**	***Haemocystidium-like* (2)**
	**Nile monitor**	*Varanus niloticus*	**4**	-	-
	**Dwarf crocodile**	*Osteolaemus tetraspis*	**7**	-	-
	**African rock python**	*Python sebae*	**6**	-	-

Historically, infections by haemosporidian parasites in ungulates were considered to be uncommon [27]; nevertheless, two haemosporidian species were previously described in African antelopes. Both were classified within the genus *Plasmodium* (*Plasmodium cephalophi* and *Plasmodium brucei*) and were first detected in Grim’s duikers (*Sylvicapra grimmia*) [28]. Therefore, the parasites identified in the present study could correspond, or could be related to these two previously described species. However, because of the absence of molecular data on these two previously described species and the lack of morphological descriptions in our study, it is impossible to draw any conclusion.

**Fig 3:**
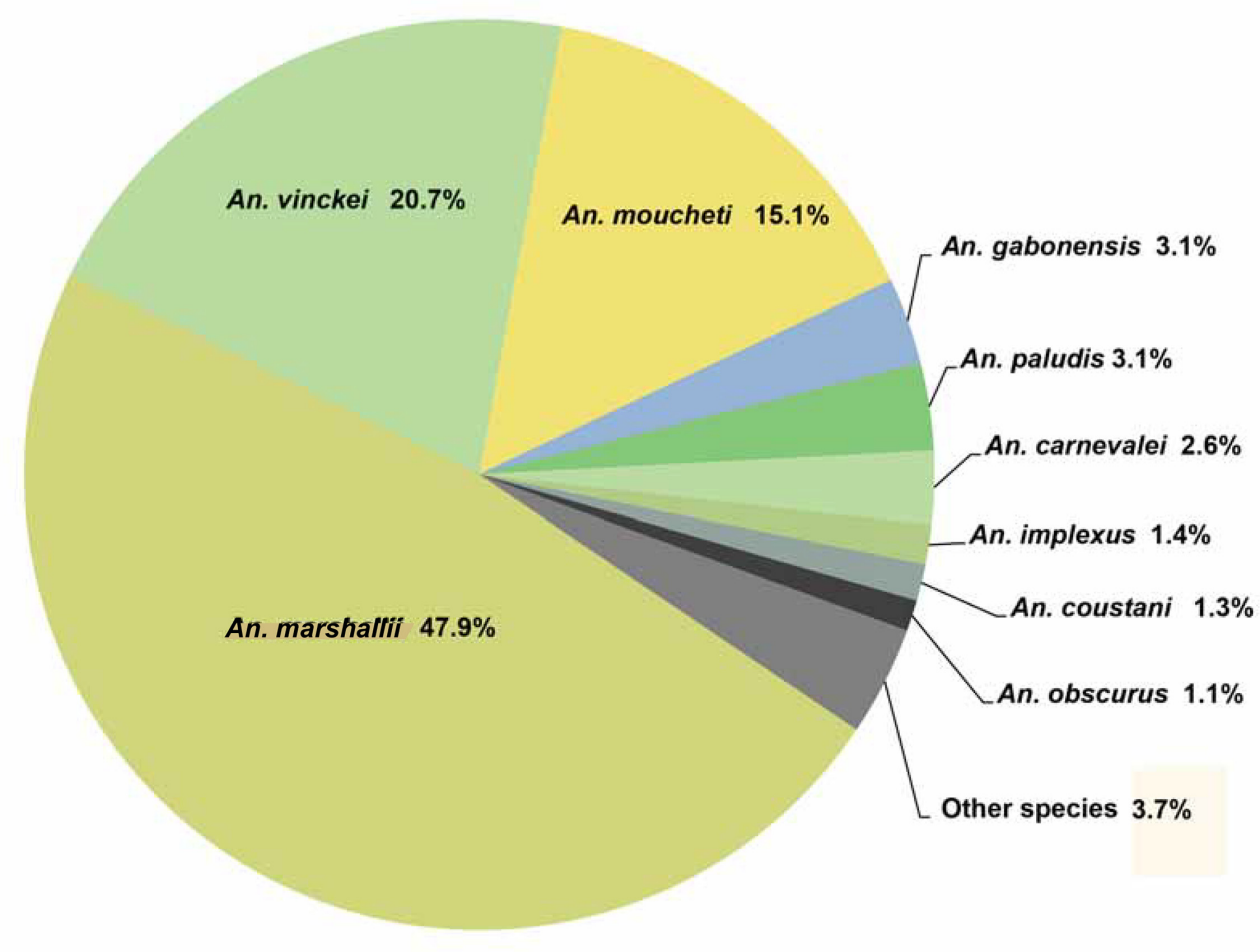
Diversity of *Anopheles* mosquitoes collected in the two wildlife reserves (La Lopé and La Lékédi) in Gabon from October 2012 to December 2013. Only species representing more than 1% of the entire population are indicated. The other species are grouped in the “other species” category.

Analysis of the molecular data from anopheline specimens collected in two forest areas of Gabon during a longitudinal survey (Fig 1 and Fig 3) indicated that they also were infected by parasites from lineages A and B (Fig 2). Specifically, we identified lineage A parasites in the DNA from the abdomen, but not from the salivary glands, of one female mosquito specimen belonging to *Anopheles gabonensis* (a species recently discovered in the Gabonese forest [29]) and in one *Anopheles vinckei* sample (Fig 2). We found parasites from lineage B most frequently (11 times) in mosquitoes belonging to six *Anopheles* species [*Anopheles carnevalei* (one specimen), *Anopheles coustani* (one specimen), *An*. *gabonensis* (four specimens), *Anopheles obscurus* (three specimens), *An*. *vinckei* (one specimen) and *An*. *sp*. (one specimen)]. Two specimens (identified as *An*. *gabonensis* and *An*. *obscurus*) harbored the parasites in the salivary glands (Fig 2). This strongly suggests that lineage B parasites can complete their life cycle within *An*. *gabonensis* and *An*. *obscurus* and produce infective forms (i.e., sporozoites that can be inoculated in a mammalian host with the saliva during blood feeding). Although we do not have any evidence about the viability of such sporozoites, we hypothesize that these two mosquito species might contribute to the transmission of lineage B parasites. Moreover, anopheline mosquitoes, but not vertebrate specimens, were infected also by two other Haemosporida lineages (C and D) that are phylogenetically related to lineages A and B (Fig 2). We found lineage C six times in *Anopheles* specimens belonging to three species: *An*. *coustani* (one specimen), *Anopheles marshallii* (three specimens) and *Anopheles moucheti* (two specimens). The identification of lineage C parasites in the salivary glands of *An*. *marshallii* (one specimen) and *An*. *moucheti* (one specimen) suggests a potential role as vectors. Conversely, we detected lineage D only in one *An*. *obscurus* specimen. For lineages C and D, the nature of their vertebrate host remains to be discovered. However, the parasites were recovered in *Anopheles* species that are known to have mainly a mammalophilic feeding behavior [30]. This suggests that mosquitoes might have acquired such parasites from mammalian hosts.

Our findings highlight several features of the ecology of these lineages. First, these parasites are not host-specific because they can infect a variety of hosts, including different species of antelopes. The propensity of ungulate haemosporidian parasites to infect different hosts was previously described [27, 31, 32] and led to the hypothesis [27] that *P*. *cephalophi* and *P*. *brucei* could be more widely distributed and possibly present also in other African antelopes. Moreover, their host range might not be restricted only to antelopes because lineage B includes parasites detected also in other orders, such as Pholidotas (pangolin) or Primates (*Cercophitecus sp*.) [20]. Whether these non-ungulate species are frequent hosts of these lineages or only accidental hosts remains to be clarified. Previous studies on African monkeys rather support the second hypothesis because only one monkey, among the several hundred individuals studied, was found to be infected by parasites belonging to lineage B [20, 21].

Second, our results suggest that some sylvan *Anopheles* species could serve as vector for these lineages. Indeed, we found that at least four *Anopheles* species (two species for lineage B and two for lineage C) might support the transmission of this group of parasites in forests. Other *Anopheles* species also were infected by these lineages, but we currently have no evidence that they serve as vectors (we detected the parasite *Cyt-b* gene sequence only in the whole body and not in salivary glands). Very little is known about the biting behavior of sylvan *Anopheles* and about the vertebrate hosts that constitute their preferred source of blood [33]. Zoophilic species, such as *An*. *vinckei* and *An*. *gabonensis*, were previously shown to transmit zoonotic *Plasmodium* parasites, including those circulating among great apes and rodents [29, 33, 34]. Our study suggests that these species also bite ungulates. This indicates that, concerning their blood meal, *An*. *vinckei* and *An*. *gabonensis* are opportunistic rather than specialized. Such a propensity to bite a wide range of hosts is probably an adaptive trait in response to temporal fluctuations of host diversity and density in forest environments. This feature could enhance the possibility for cross-species transfer of parasites and could explain the parasite propensity to infect different host species. In addition, the panel of *Anopheles* species infected by these newly described haemosporidian lineages encompassed mosquito species with a well-known anthropophilic feeding behavior (sometimes very pronounced, such as in *An*. *marshallii* or *An*. *mouchetii* [33]).

Finally phylogenetic analysis of the relationship between the *Cyt-b* sequences of the haemosporidian parasites obtained in our study and reference sequences (S1 File) indicated that the phylogenetic position of the four newly described haemosporidian lineages was closer to sauropsid *Plasmodium* and *Polychromophilus* (bat parasites) than to other mammalian parasites (Fig 2, Figure A in S1 File, and Figure B in S1 File). According to the current Haemosporida classification, all species infecting mammals belong to different genera within the Plasmodiidae family [2]. However, molecular data are lacking for several genera identified in bats or flying squirrels (e.g., *Biguetiella*, *Dionisia*, *Bioccala* and *Rayella*) and they were never included in modern phylogenies. Previous molecular-based studies proposed that most Plasmodiidae mammalian parasites (i.e., the genera *Plasmodium* and *Hepatocystis*) belong to a single monophyletic clade and are transmitted by anopheles (e.g., [24–26]), with the exception of some bat parasites (genera *Polychromophilus* and *Nycteria*). It was suggested that parasites of the genus *Polychromophilus* in bats could be the result of a secondary invasion of mammals and that they derived from avian or reptile parasites (sauropsid *Plasmodium*) and were transmitted by bat flies [35]. The origin of *Nycteria* parasites is less clear and needs to be further investigated. Particularly, it is unclear whether they represent another case of host switch or an ancient mammalian *Plasmodium* lineage. Similarly, the origin of the four clades described in this study is uncertain. Indeed, depending on the phylogenetic analyses (e.g., different rooting strategies) and reference sequences we used, the position of the four clades relatively to sauropsid *Plasmodium* or to *Polychromophilus* parasites changed (i.e., closer to sauropsid *Plasmodium* or to *Polychromophilus* species; data not shown). Therefore, it remains to be determined whether these four clades are the result of a host switch from sauropsid *Plasmodium* or whether they are more related to *Polychromophilus* parasites. Another remaining question concerns the roles played by the vertebrate hosts and their vectors in their evolution and diversification.

Besides these four lineages discovered in antelopes and/or sylvan mosquitoes, we identified two additional lineages in bushmeat samples: one in a bird sample and the other in two tortoise specimens. The bird lineage was related to parasites of the *Haemoproteus* genus [36] and the turtle lineage was related to parasites of the *Haemocystidium* genus [2, 36]. More data on these groups of vertebrates are required to determine whether these last two lineages are new [2, 25, 36]. Indeed, the current data and those available in the literature are too scarce to conclude with confidence.

## Conclusion

In this study, we identified four new haemosporidian molecular lineages that belong to the Plasmodiidae family and that might be transmitted by *Anopheles* mosquitoes. Concerning their vertebrate hosts, two of the lineages (A and B) can infect several mammalian species. The vertebrate hosts of the other two lineages (C and D) remain to be identified. None of these four lineages could be matched against reference sequences, although several species of haemosporidian parasites were previously described in African antelopes, but only based on their morphological features. As a consequence, and in agreement with other authors (e.g., [2]), we think that it is now crucial to rapidly undertake a complete re-evaluation of the taxonomy of these protozoa in the different groups of vertebrates with a particular attention to the genus *Plasmodium*. Indeed, this genus includes some species that are more genetically distant between them than they are with species belonging to other genera. Ideally, this re-evaluation should be done using the different criteria of phenotypic similarities, the concept of biological species and, most importantly, the concept of phylogenetic species [2, 10]. Such a re-evaluation is critical to our understanding of host-pathogen evolution in this group of parasites.

## Material and Methods

### Wildlife screening

To analyze the diversity of haemosporidian parasites circulating among wild vertebrates in Gabon (Central Africa), we screened several bushmeat samples from wild animal species collected by the CIRMF (Centre International de Recherches Médicales de Franceville) in eight provinces of Gabon (Fig 1) between 2009 and 2013. Most bushmeat samples (n = 347) were obtained from officers of the provincial direction of the Gabon Ministry of Water Affairs and Forestry after their seizure because of illegal hunting and 145 from bushmeat salesmen during hunting periods. Species identification was performed by visual inspection according to the Kingdom Field Guide to African Mammals [37] and the “Guide des Reptiles du Gabon” [38]. Biological samples of interest (whole blood, liver or spleen) were taken and kept in liquid nitrogen until delivery to the CIRMF where they were stored at -80°C for molecular analyses. For each selected animal, total DNA was extracted from approximately 200μl of blood or 100mg of liver/spleen according to the procedures described in [21] and [39], respectively.

#### Ethic statement

The study was conducted in Gabon outside protected areas. All samples were collected from dead animals only (bushmeat). Some animal carcasses confiscated from poachers by Gabonese authorities (Fauna and Hunting Department, Ministry of Water Affairs and Forestry, Environment and Sustainable development of Gabon) belonged to protected species (e.g., water chevrotain). All samples obtained from bushmeat salesmen were collected only during the authorized hunting season of non-protected species and only in public markets. No money was given to the salesmen in exchange of the pieces of tissues from the animals. All samples were collected for a CIRMF project aiming at identifying the natural reservoir of Ebola virus. New samples were not collected for this study. All work was carried out with the authorization from the Gabonese Ministry of Water Affairs and Forestry (Département de la Faune et de la Chasse—Authorization N°005/MEFEDD /SG/DGEF/PEFWN, N°001/MEFEDD/SG/DGEF/PEENY, N°108/MEFEDD/SG/DGEF/ PEFOL and N°016/MEFEDD/SG/DGEF/PEFMO) and the Gabonese Ministry of Higher Education, Scientific Research and Innovation (Centre National de la Recherche Scientifique et Technique—Authorization N°AR0031/09 /MENESRESI/ CENAREST/CG/CST/CSAR).

### Mosquito sampling

Mosquitoes were sampled in two wildlife reserves in Gabon: the Lopé National Park (Ogooué-Ivindo province) and the private park of La Lékédi, near Bakoumba (Haut-Ogooué province). At both sites (Fig 1), a longitudinal survey of sylvan *Anopheles* was carried out using CDC light traps placed in several sites of the forest between 5pm to 7am from October 2012 to December 2013. Mosquitoes were morphologically identified using identification keys [40]. Salivary glands of females *Anopheles* were separated from the rest of the body. All samples were stored in liquid nitrogen and transferred to the CIRMF where they were kept at -80°C. In total, 2,184 females of *Anopheles* were collected that belonged to 17 species. *An*. *marshallii* (47.9%), *An*. *vinckei* (20.7%) and *An*. *moucheti* (15.1%) mosquitoes constituted most of the captures. The other *Anopheles* (14%) belonged to other species and only 2.3% of *Anopheles* could not be identified (Fig 3).

### Molecular analyses

Total DNA was extracted from bushmeat tissues, mosquito bodies and salivary glands with the DNeasy Blood and Tissue Kit (Qiagen) and used as template for the detection of haemosporidian parasites according to a previously described protocol [16] based on PCR amplification and sequencing of a portion of the parasite *Cyt-b* gene. All amplified products (10 μl) were run on 1.5% agarose gels in TBE buffer. Amplicons were then sequenced by Eurofins MWG (Germany). The sequences reported in this study were deposited in GenBank under the following accession numbers KT367817 to KT367865.

For species identification, each sequence was compared to a list of reference sequences obtained from GenBank (http://www.ncbi.nlm.gov/) (S1 File). Because amplicons with chimeric sequences can form during PCR amplification of DNA from samples with multiple infections, similarity plot analyses were performed on the nucleotide alignments generated with the new and all reference sequences using the SIMPLOT package version 2.5 [41] and a sliding window of 80 nucleotides (nt) moved in steps of 10 nt. Once verified that the amplicons did not contain chimeric sequences, phylogenetic analyses were done after multiple alignments of the obtained partial *Cyt-b* sequences (757 nucleotides) and of the Genbank reference sequences using ClustalW (v 1.8.1 in BioEdit v.7.0.9.0. Software) [42]. It was previously shown that enough phylogenetic data can be extracted from *Cyt-b* sequences to study the phylogenetic relationships between haemosporidian parasites and to recover major clades [43, 44]. Maximum Likelihood (ML) methods were used for tree construction [16]. The best-fitting ML model based on the Akaike Information Criterion was GTR (General Time Reversible) + Gamma + I (invariant sites), as determined using ModelTest [45]. The highest-likelihood DNA tree and the corresponding bootstrap support values were obtained by using PhyML [46, 47] (freely available at the ATGC bioinformatics platform http://www.atgc-montpellier.fr/) using NNI (Nearest Neighbor Interchange) + SPR (Subtree Pruning Regrafting) branch swapping and 100 bootstrap replicates. Recently, the choice of outgroup to use to root the tree of haemosporidian species has been a matter of debate. In our study, ML trees were rooted: (i) using *Toxoplasma* spp. and *Eimeria* spp.; and (ii) using *Leucocytozoon* spp (S1 File). Finally, BEAST [46] was used to implement a Bayesian outgroup-free method to construct the tree under a Yule tree process and by assuming a log-normal relaxed molecular clock and the GTR substitution model of evolution, with gamma distributed rates (four categories) and including a proportion of invariant sites (GTR + G + I). Three independent runs were performed for 30 million generations sampled every 5,000 generations (burn-in). The traces and the effective sample size (ESS; all exceeded 250) were checked using TRACER v. 1.6.0 [48], to evaluate the Markov chain Monte Carlo convergence. The three independent runs were combined with LOG-COMBINER v.1.8.2. [49] and the phylogeny was determined using TREEANNOTATOR v.1.8.2 [49] and FigTree v.1.4.2 [50] for visualization. One advantage of estimating a phylogeny using a relaxed clock is that an estimate of the position of the tree root can be obtained.

## Supporting Information

S1 File Phylogenetic trees and Accession numbers of all sequences. The trees give the relationship between our sequences and others sequences of genbank. Phylogenetic tree was rooted using *Leucocytozoon sp*p (Figure A). Phylogenetic tree was rooted using *Eimeria spp* (Figure B). And one table gives all accession numbers of sequences used in our study (Table A).
(PDF)
