# Supplementary material for: Haemosporidian Parasites of Antelopes and Other Vertebrates from Gabon, Central Africa
Source: PLoS One. 2016 Feb 10;11(2):e0148958. doi: 10.1371/journal.pone.0148958 (PMC4749209; doi:10.1371/journal.pone.0148958)
Supplement: S1 File — The trees give the relationship between our sequences and others sequences of genbank. Phylogenetic tree was rooted using Leucocytozoon spp (Figure A). Phylogenetic tree was rooted using Eimeria spp (Figure B). And one table gives all accession numbers of sequences used in our study (Table A). (PDF) [file pone.0148958.s001.pdf]

Table A. ***Cyt-b*** **sequences of parasites recovered in this study and those used as references for phylogenetic analyses and their Genbank accession numbers.**

Accession numbers	Isolates name	Host
**HQ702482**	*Eimeria necatrix*	Fowl
**HQ173882**	*Eimeria magna*	*Rabbit*
**HQ173892**	*Eimeria vejdovskyi*	*Rabbit*
**AF023246**	*Toxoplasma_gondii*	-
**AY099053**	*P. giganteum*	*Bird*
**AY099054**	*P. atheruri*	*Rodent*
**AY099055**	*P. azurophilum*	*Bird*
**AY733090**	*P. relictum*	*Bird*
**DQ414658**	*P. yoelii_killicki*	*Rodent*
**FJ168561**	*Parahaemoproteus_vireonis*	*bird*
**FJ168565**	*Hepatocystis_sp*	*Bird*
**GQ141574**	*Plasmodium sp._bird_sp.12*	*Bird*
**GQ141581**	*Parahaemoproteus_sp._bird_sp.17*	*Bird*
**GQ141585**	*Parahaemoproteus_sp._bird_sp.19*	*Bird*
**GU252012**	*P. sp._GD2_GD201*	*Bird*
**GU252027**	*Plasmodium sp._JA7_J725*	*Bird*
**AY099045**	*Haemoproteus_majoris*	*Bird*
**HM222472**	*Haemoproteus_sp*	*Bird*
**HM222485**	*Plasmodium_sp*	*Bird*
**HM222486**	*Haemoproteus_sp*	*Bird*
**HM222487**	*Haemoproteus_sp*	*Bird*
**HQ712051**	*P. atheruri*	*Rodent*
**JQ070884**	*Hepatocystis_sp._S2138*	*C. nictitans*
**JQ070951**	*Hepatocystis_sp._AA-201_b-like*	*C. nictitans*
**JQ070956**	*Hepatocystis_sp._AA-2012*	*C. nictitans*
**JQ995287**	*Polychromophilus_sp._LD-2012*	*Bat*
**KF049492**	*E15-Podocnemis expansa*	*Turtle*
**KF049495**	*E24-Podocnemis expansa*	*Turtle*
**KF049506**	*U65-Podocnemis unifilis*	*Turtle*
**KF159681**	*Polychromophilus sp._Min_vil_G3*	*Bat*
**KJ131277**	*Polychromophilus melanipherus*	*Bat*
**KF159699**	*Polychromophilus sp._Min_vil_G3_2*	*Bat*
**HM055585**	*Polychromophilus murinus*	*Bat*
**KF159714**	*Polychromophilus sp._Pip_gran_G3*	
**KF159690**	*Nycteria_sp._R_lan_G3_1*	*Bat*
**KF159700**	*Polychromophilus_sp._Neo_cap_G3*	*Bat*
**KF159720**	*Nycteria_sp_R_alc_C9_1*	*Bat*
**KJ700853**	*P. vinckei_isolate_1*	*Rodent*
**KJ700854**	*P. vinckei_isolate_2*	*Rodent*
**KM598212**	*P. sp._lineage_JA01*	*Bird*
**NC_012450**	*Leucocytozoon_majoris*	*Bird*
**FJ168563**	*Leucocytozoon_majoris*	*Bird*
**AY099063**	*Leucocytozoon dubreuli*	*Bird*
**AB299369**	*Leucocytozoon sabrazesi*	*Bird*
**AB302215**	*Leucocytozoon caulleryi*	*Bird*
**HM235081**	*P. adleri*	*Gorilla*
**KP875474**	*P. billcollinsi*	*Chimpanzee*
**HM235065**	*P. blacklocki*	*gorilla*
**JF923762**	*P. praefalciparum*	*Cercopithecus nictitans*
**FJ895307**	*P. gaboni*	*Chimpanzee*
**JF923751**	*P. gonderi*	*Mandrill*
**HM000110**	*P. malariae*	*Chimpanzee*
**GU723548**	*P. ovale*	*Human*
**KP875479**	*P. reichenowi*	*Chimpanzee*
**JF923753**	*P. sp._DAJ-2004*	*mandrill*

**KF591834**	*P. vivax*	*Human*
**KF159674**	*P. cyclopsi*	*Bat*
**KF159671**	*P. voltaicum*	*Bat*
**AB444126**	*P. cynomolgi*	-
**AF069612**	*P. gallinaceum*	*Bird*
**JQ345504**	*P. knowlesi*	*Human*
**NC_012447**	*Parahaemoproteus vireonis*	*Vireogilvus*
**KT367840**	*WN-622_Cephalophus monticola*	Antelope
**KT367833**	*HO-13_ Cephalophus monticola*	Antelope
**KT367822**	*OL-122_Cephalophus_monticola*	Antelope
**KT367835**	*OM-449_Cephalophus_monticola*	Antelope
**KT367828**	*OL-100_Cephalophus_monticola*	Antelope
**KT367830**	*OL-131_Cephalophus_callipygus*	Antelope
**KT367841**	*NG-235_Cephalophus_monticola*	Antelope
**KT367819**	*HO-11_Cephalophus_nigrifons*	Antelope
**KT367839**	*MO-309_Cephalophus_monticola*	Antelope
**KT367851**	*LOP_An._vinckei178*	Anophele
**KT367852**	*LOP_An._gabonensis3*	Anophele
**KT367837**	*OL-149_Cephalophus_monticola*	Antelope
**KT367832**	*OL-115-Cephalophus_monticola*	Antelope
**KT367834**	*MO-278_Cephalophus_monticola*	Antelope
**KT367817**	*OI-79_Cephalophus_dorsalis*	Antelope
**KT367821**	*NG-215_Cephalophus_dorsalis*	Antelope
**KT367842**	*OI-49_Cephalophus_dorsalis*	Antelope
**KT367838**	*NY-195_Cephalophus_dorsalis*	Antelope
**KT367823**	*OM-22_Cephalophus_dorsalis*	Antelope
**KT367826**	*OI-90_Cephalophus_dorsalis*	Antelope
**KT367820**	*OM-14_Cephalophus_dorsalis*	Antelope
**KT367829**	*WN-384_Cephalophus_dorsalis*	Antelope
**KT367825**	*NG-227_Cephalophus_dorsalis*	Antelope
**KT367836**	*HO-13_Cephalophus_monticola*	Antelope
**KT367831**	*OL-120_Cephalophus_dorsalis*	Antelope
**KT367824**	*OL-NG226_Cephalophus_monticola*	Antelope
**KT367827**	*HO-A6_Cephalophus_monticola*	Antelope
**KT367818**	*OI-52_Pangolin*	*Pangolin*
**KT367865**	*LOP_An._gabonensisSG279*	Anophele
**KT367853**	*LOP_An._gabonensis279*	Anophele
**KT367855**	*LEK_An_coustani1404*	Anophele
**KT367847**	*LOP_An._carnevalei*	Anophele
**KT367860**	*LEK_An._gabonensis*	Anophele
**KT367849**	*LOP_An._obscurus78*	Anophele
**KT367863**	*LOP_An._obscurusSG78*	Anophele
**KT367848**	*LOP_An._obscurus2*	Anophele
**KT367850**	*LOP_An._sp177*	Anophele
**KT367861**	*LEK_An._gabonensis2*	Anophele
**KT367854**	*LEK_ An._vinckei178*	Anophele
**KT367858**	*LEK_An._marshallii1598*	Anophele
**KT367859**	*LEK_An._moucheti1275*	Anophele
**KT367856**	*LEK_An._coustani1338*	Anophele
**KT367864**	*LEK_An._mouchetiSG1407*	Anophele
**KT367862**	*LEK_An._marshalliiSG1682*	Anophele
**KT367857**	*LEK_An._marshallii1682*	Anophele
**KT367846**	*LEK_An._obscurus2*	Anophele
**KT367843**	*HO-9_Kinixys_erosa*	*Turtle*
**KT367844**	*NG-238_Kinixys_erosa*	*Turtle*
**KT367845**	*NG-277_Ceratogymna atrata*	*Bird*

Figure legend

Figures

Phylogenetic relationships between the *Cyt-b* sequences obtained in our study (in brown) and reference sequences from existing databases. The trees were built based on 757 bp long cytochrome B (*Cyt-b*) sequences using Maximum Likelihood methods. Branch colors indicate different groups of vertebrates. A) the maximum likelihood tree was rooted using *Leucocytozoon* spp.. B) the tree was rooted using *Eimeria* spp. The names of our isolates include: 1) the abbreviation of the sampling site (LOP: La Lopé and LEK: Lékédi) or province (OL: Ogooue-Lolo; OM: Ogooue-Maritime, OI: Ogooue-Ivindo, NG: Ngounie; NY: Nyanga; HO: Haut-Ogooue; MO: Moyen-Ogooue; WN: Woleu-Ntem), 2) the sample number and 3) the name of the host species in which it was found (vertebrate host or anopheles) (for instance, OL115-*Cepholophus monticola*), 4) SG if the parasite was detected in the salivary glands (for anopheles only).

Figure A

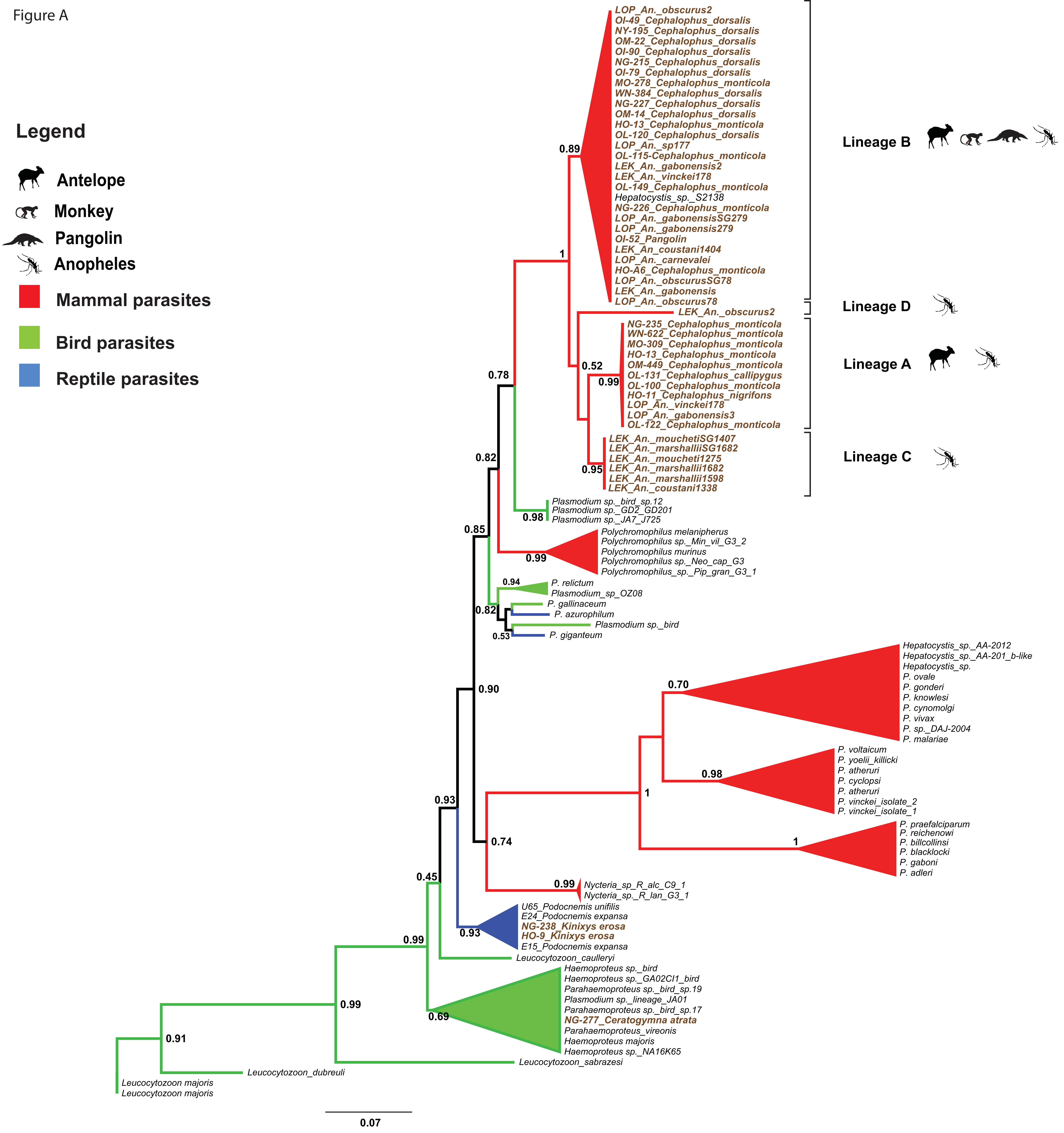
Legend
Antelope
Monkey
Pangolin
Anopheles
Mammal parasites
Bird parasites
Reptile parasites
Lineage B
Lineage D
Lineage A
Lineage C
LOP_An._obscurus2
OI-49_Cephalophus_dorsalis
NY-195_Cephalophus_dorsalis
OM-22_Cephalophus_dorsalis
OI-90_Cephalophus_dorsalis
NG-215_Cephalophus_dorsalis
OI-79_Cephalophus_dorsalis
MO-278_Cephalophus_monticola
WN-384_Cephalophus_dorsalis
NG-227_Cephalophus_dorsalis
OM-14_Cephalophus_dorsalis
HO-13_Cephalophus_monticola
OL-120_Cephalophus_dorsalis
LOP_An._sp177
OL-115-Cephalophus_monticola
LEK_An._gabonensis2
LEK_An._vinckei178
OL-149_Cephalophus_monticola
Hepatocystis_sp._S2138
NG-226_Cephalophus_monticola
LOP_An._gabonensisSG279
LOP_An._gabonensis279
OI-52_Pangolin
LEK_An_coustani1404
LOP_An._carnevalei
HO-A6_Cephalophus_monticola
LOP_An._obscurusSG78
LEK_An._gabonensis
LOP_An._obscurus78
LEK_An._obscurus2
NG-235_Cephalophus_monticola
WN-622_Cephalophus_monticola
MO-309_Cephalophus_monticola
HO-13_Cephalophus_monticola
OM-449_Cephalophus_monticola
OL-131_Cephalophus_callipygus
OL-100_Cephalophus_monticola
HO-11_Cephalophus_nigrifons
LOP_An._vinckei178
LOP_An._gabonensis3
OL-122_Cephalophus_monticola
LEK_An._mouchetiSG1407
LEK_An._marshalliiSG1682
LEK_An._moucheti1275
LEK_An._marshallii1682
LEK_An._marshallii1598
LEK_An._coustani1338
Plasmodium sp._bird_sp.12
Plasmodium sp._GD2_GD201
Plasmodium sp._JA7_J725
Polychromophilus melanipherus
Polychromophilus sp._Min_vil_G3_2
Polychromophilus murinus
Polychromophilus sp._Neo_cap_G3
Polychromophilus_sp._Pip_gran_G3_1
P. relictum
Plasmodium_sp_OZ08
P. gallinaceum
P. azurophilum
Plasmodium sp._bird
P. giganteum
Hepatocystis_sp._AA-2012
Hepatocystis_sp._AA-201_b-like
Hepatocystis_sp.
P. ovale
P. gonderi
P. knowlesi
P. cynomolgi
P. vivax
P. sp._DAJ-2004
P. malariae
P. voltaicum
P. yoelii_killicki
P. atheruri
P. cyclopsi
P. atheruri
P. vinckei_isolate_2
P. vinckei_isolate_1
P. praefalciparum
P. reichenowi
P. billcollinsi
P. blacklocki
P. gaboni
P. adleri
Nycteria_sp_R_alc_C9_1
Nycteria_sp._R_lan_G3_1
U65_Podocnemis unifilis
E24_Podocnemis expansa
NG-238_Kinixys erosa
HO-9_Kinixys erosa
E15_Podocnemis expansa
Leucocytozoon_caulleryi
Haemoproteus sp._bird
Haemoproteus sp._GA02Cl1_bird
Parahaemoproteus sp._bird_sp.19
Plasmodium sp._lineage_JA01
Parahaemoproteus sp._bird_sp.17
NG-277_Ceratogymna atrata
Parahaemoproteus_vireonis
Haemoproteus majoris
Haemoproteus sp._NA16K65
Leucocytozoon_sabrazesi
Leucocytozoon_dubreuli
Leucocytozoon majoris
Leucocytozoon majoris
0.89
1
0.52
0.99
0.95
0.78
0.82
0.98
0.85
0.99
0.94
0.82
0.53
0.70
0.90
0.98
0.93
1
0.74
1
0.99
0.45
0.93
0.99
0.99
0.69
0.91
0.07

Figure B

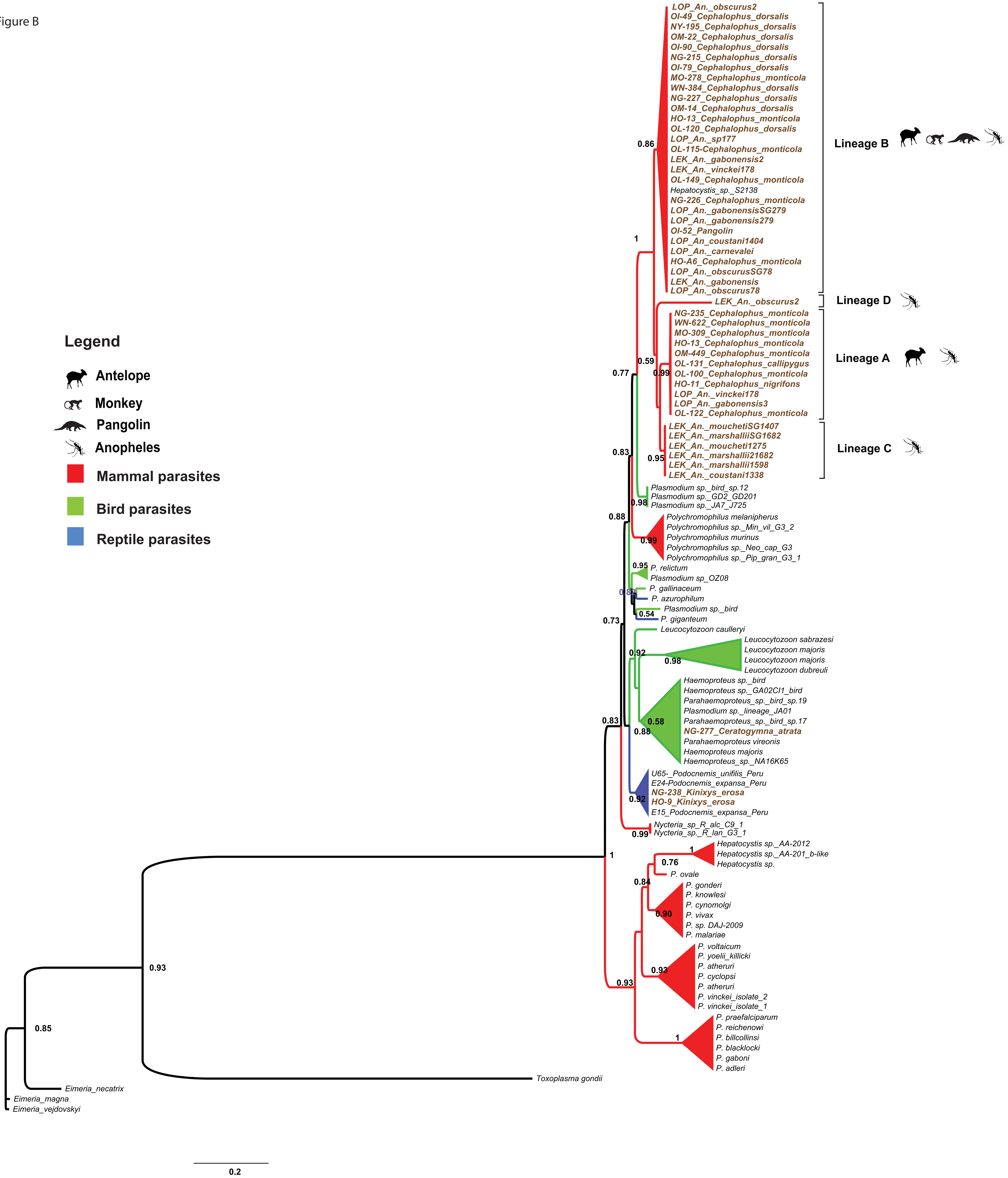

LOP_An._obscurus2
OI-49_Cephalophus_dorsalis
NY-195_Cephalophus_dorsalis
OM-22_Cephalophus_dorsalis
OI-90_Cephalophus_dorsalis
NG-215_Cephalophus_dorsalis
OI-79_Cephalophus_dorsalis
MO-278_Cephalophus_monticola
WN-384_Cephalophus_dorsalis
NG-227_Cephalophus_dorsalis
OM-14_Cephalophus_dorsalis
HO-13_Cephalophus_monticola
OL-120_Cephalophus_dorsalis
LOP_An._sp177
OL-115-Cephalophus_monticola
LEK_An._gabonensis2
LEK_An._vinckei178
OL-149_Cephalophus_monticola
Hepatocystis_sp._S2138
NG-226_Cephalophus_monticola
LOP_An._gabonensisSG279
LOP_An._gabonensis279
OI-52_Pangolin
LOP_An_coustani1404
LOP_An._carnevalei
HO-A6_Cephalophus_monticola
LOP_An._obscurusSG78
LEK_An._gabonensis
LOP_An._obscurus78
Lineage B
LEK_An._obscurus2
Lineage D
NG-235_Cephalophus_monticola
WN-622_Cephalophus_monticola
MO-309_Cephalophus_monticola
HO-13_Cephalophus_monticola
OM-449_Cephalophus_monticola
OL-131_Cephalophus_callipygus
OL-100_Cephalophus_monticola
HO-11_Cephalophus_nigrifons
LOP_An._vinckei178
LOP_An._gabonensis3
OL-122_Cephalophus_monticola
Lineage A
LEK_An._mouchetiSG1407
LEK_An._marshalliiSG1682
LEK_An._moucheti1275
LEK_An._marshallii21682
LEK_An._marshallii1598
LEK_An._coustani1338
Lineage C
Legend
Antelope
Monkey
Pangolin
Anopheles
Mammal parasites
Bird parasites
Reptile parasites
0.86
1
0.59
0.99
0.77
0.95
0.83
Plasmodium sp._bird_sp.12
Plasmodium sp._GD2_GD201
Plasmodium sp._JA7_J725
0.98
0.88
Polychromophilus melanipherus
Polychromophilus sp._Min_vil_G3_2
Polychromophilus murinus
Polychromophilus sp._Neo_cap_G3
Polychromophilus sp._Pip_gran_G3_1
0.99
0.95
P. relictum
Plasmodium sp_OZ08
P. gallinaceum
P. azurophilum
Plasmodium sp._bird
0.54
P. giganteum
0.73
Leucocytozoon caulleryi
Leucocytozoon sabrazesi
Leucocytozoon majoris
Leucocytozoon majoris
Leucocytozoon dubreuli
0.92
0.98
Haemoproteus sp._bird
Haemoproteus sp._GA02CI1_bird
Parahaemoproteus_sp._bird_sp.19
Plasmodium sp._lineage_JA01
Parahaemoproteus_sp._bird_sp.17
NG-277_Ceratogymna_atrata
Parahaemoproteus vireonis
Haemoproteus majoris
Haemoproteus_sp._NA16K65
0.83
0.58
0.88
U65-_Podocnemis_unifilis_Peru
E24-Podocnemis_expansa_Peru
NG-238_Kinixys_erosa
HO-9_Kinixys_erosa
E15_Podocnemis_expansa_Peru
0.92
Nycteria_sp_R_alc_C9_1
Nycteria_sp._R_lan_G3_1
0.99
1
Hepatocystis sp._AA-2012
Hepatocystis sp._AA-201_b-like
Hepatocystis sp.
1
0.76
P. ovale
0.84
P. gonderi
P. knowlesi
P. cynomolgi
P. vivax
P. sp. DAJ-2009
P. malariae
0.90
P. voltaicum
P. yoelii_killicki
P. atheruri
P. cyclopsi
P. atheruri
P. vinckei_isolate_2
P. vinckei_isolate_1
0.93
0.93
P. praefalciparum
P. reichenowi
P. billcollinsi
P. blacklocki
P. gaboni
P. adleri
1
0.93
0.85
Toxoplasma gondii
Eimeria_necatrix
Eimeria_magna
Eimeria_vejdovskyi
0.2
